# AutoFOCUS: A mindfulness-based intervention for caregivers of autologous hematopoietic stem cell transplant recipients

**DOI:** 10.1017/S1478951525100485

**Published:** 2025-08-22

**Authors:** Valerie Yepez, Min-Jeong Yang, Sierra Washington, Sarah Jones, Ranjita Poudel, Joseph Pidala, Marilyn Horta, Christine Vinci

**Affiliations:** 1 Philadelphia College of Osteopathic Medicine South Georgia, Moultrie, GA, USA; 2 Department of Health Outcomes and Behavior, H. Lee Moffitt Cancer Center, Tampa, FL, USA; 3 Department of Blood and Marrow Transplantation and Cellular Immunotherapy, H. Lee Moffitt Cancer Center, Tampa, FL, USA; 4 Department of Oncological Science, College of Medicine, University of South Florida, Tampa, FL, USA

**Keywords:** Autologous hematopoietic stem cell transplant, telehealth, mental health, burden, meditation

## Abstract

**Objectives:**

Autologous hematopoietic stem cell transplant (HCT) cancer caregivers experience significant burden and stress with limited tailored resources. Mindfulness interventions hold promise in alleviating caregiver distress. Predicated on our previous work with allogeneic HCT caregivers, this single-arm trial tested the feasibility and acceptability of a modified mindfulness-based intervention, AutoFOCUS, among autologous HCT caregivers.

**Methods:**

Participants received the 6-session AutoFOCUS face-to-face via telehealth, with assessments at baseline, end of treatment, and 1-month post-treatment. Feasibility was assessed through recruitment, retention, and session attendance, and acceptability was measured via satisfaction and intent to continue using skills learned. Exploratory outcome measures included distress, anxiety, perceived stress, affect, and post-traumatic growth. Data from the smartphone app that supplemented the face-to-face component of the intervention were collected. In-depth interviews gathered participant feedback.

**Results:**

Twenty-six caregivers (mean age = 57.7 years, 89% female) were enrolled and 19 completed at least 4 sessions, 14 completed all 6 sessions, and 22 completed the 1-month follow-up. High satisfaction (M = 3.56/4; SD = 0.43) and intent to utilize the skills learned in the future (M = 8.58/10; SD = 1.81/4) were reported. Significant reductions in distress (*p* < .001, (effect sizes [ES]) = 0.99), anxiety (*p* = .032, [ES] = 0.53), perceived stress (*p* = .035, [ES] = 0.52), and negative affect (*p* = .008, [ES] = 0.69) were reported, along with a significant increase in post-traumatic growth (*p* = .009, [ES] = 0.67) from baseline to end of treatment. App use was moderate. Interview results highlighted positive perceptions and supported quantitative results.

**Significance of results:**

AutoFOCUS was feasible and acceptable. Future studies should explore the efficacy of this treatment on a larger scale with a comparison condition.

## Introduction

Caregivers of hematopoietic stem cell transplant (HCT) patients often report diminished well-being, uncertainty about the future, and difficulty adapting to their new role post-transplant while also experiencing elevated symptoms of anxiety and depression compared to non-caregivers (Boyle et al. 2000; Jim et al. 2014; Simoneau et al. 2013; Stetz et al. 1996; Wilson et al. 2009). HCT caregiving involves significant responsibilities that disrupt routines and can lead to interpersonal difficulties, with the potential to negatively impact both caregiver and patient well-being (Ankuda et al. 2017; Bishop et al. 2007; Boyle et al. 2000; Gemmill et al. 2011; Grimm et al. 2000; Hochhausen et al. 2007; Jacobsen et al. 2002; Kettmann and Altmaier 2008; Rini et al. 2011; Stetz et al. 1996; Wulff-Burchfield et al. 2013). Adequate caregiver support is crucial for patient recovery. Yet, resources for HCT caregivers are often limited to hospital-based support groups and general stress management services, posing logistical challenges for HCT caregivers in the context of patient care.

Patients can receive either autologous (i.e., using their own hematopoietic cells) or allogeneic (i.e., using donor hematopoietic cells) transplants. Caregiving for autologous transplant patients differs from allogeneic patients due to distinct disease indications and treatment trajectories (Martino et al. 2021; Meehan et al. 2006). Caregivers of autologous transplant patients often face intense but relatively short-term caregiving demands concentrated around the transplant and immediate recovery period. In contrast, caregivers of allogeneic transplant patients typically encounter longer, less predictable caregiving trajectories involving risks of graft-versus-host disease, for example, that are distinct from autologous transplants (El-Jawahri et al. 2020; Martino et al. 2021). Autologous patients also have different diagnoses and treatment histories than allogeneic patients (Martino et al. 2021), and the risk for relapse post-transplant is usually higher with autologous transplants (Champlin 2003). Autologous transplants can occur in either an inpatient or outpatient setting (whereas allogeneic are all inpatient), impacting daily caregiver involvement. Outpatient caregivers often begin caregiving duties early in the treatment course, during chemotherapy, while inpatient caregivers provide full-time support through post-discharge. These differences underscore the importance of providing tailored support for autologous transplant caregivers to address their unique needs.

To our knowledge, 2 studies to date have tested a behavioral intervention for HCT caregivers that included autologous caregivers (El-Jawahri et al. 2020; McAndrew et al. 2025). The first study, by El-Jawahri et al. (2020), examined the efficacy of a 6-session intervention that used cognitive behavioral strategies delivered to both autologous and allogeneic caregivers, with promising results (e.g., improved quality of life, decreased burden). More recently, McAndrew et al. (2025) evaluated a video conference-delivered intervention tailored for caregivers of autologous transplant patients, further contributing to this emerging area of research. We posit that a mindfulness-based intervention (MBI) may address an existing limitation of cognitive behavioral therapy (i.e., the traditional view that distress is related to maladaptive and irrational cognitions), such that an MBI may provide even more benefit for this unique caregiving population (O’Toole et al. 2017).

MBIs can potentially alleviate burden and aid in stress management among HCT caregivers by promoting (1) awareness of ongoing experiences (e.g., physical, cognitive, affective), (2) cognitive flexibility to adopt new coping strategies, (3) improved emotion regulation, and (4) increased acceptance of the present moment (Carmody et al. 2009; Creswell and Lindsay 2014; Grabovac et al. 2011; Hölzel et al. 2011). Recognizing the need to reduce HCT caregiver distress to improve patient outcomes, our team developed and pilot-tested a 6-session MBI for stress management – Focusing On mindfulness for Caregivers Under Stress (FOCUS) – among caregivers of allogeneic HCT patients (Vinci et al. 2020) through a systematic process (Vinci et al. 2018). We acknowledge that there exists a separate FOCUS intervention, developed by Dr. Northouse and colleagues, which addresses the patient/caregiver dyad and quality of life for cancer patients and their caregivers through family involvement, symptom management, and coping skills training (Northouse et al. 2013). The FOCUS intervention described in the present study is distinct in content and structure, focusing specifically on mindfulness-based strategies to support caregivers during the HCT process. Our single-arm pilot study demonstrated that FOCUS was highly feasible and acceptable among 21 allogeneic HCT caregivers, with significant decreases in negative affect and increases in mindfulness, post-traumatic growth, and mental health symptoms observed with medium to large effect sizes (Vinci et al. 2020). To address the unique needs of autologous caregivers, we modified FOCUS (i.e., AutoFOCUS) and present pilot findings here.

In the current single-arm pilot study, FOCUS was modified to suit autologous caregivers by tailoring the delivery schedule based on the timing of the patient’s transplant and delivery modality; making minor modifications to the content; and supplementing face-to-face treatment with a smartphone app containing meditations and mindfulness strategies. The study involved weekly, 45-min face-to-face telehealth sessions (via Zoom) with a trained facilitator for 6 consecutive weeks, along with daily mindfulness strategies and a daily assessment administered through a smartphone app. Given that ongoing daily practice is important for skill development in mindfulness interventions (Creswell and Lindsay 2014), we supplemented live sessions with a smartphone app to facilitate practice adherence, consistent with prior work demonstrating that mobile technology can enhance engagement with behavioral interventions (Goldberg et al. 2022). The primary aim of the study was to examine the feasibility (i.e., recruitment, retention, and session attendance) and acceptability (e.g., participant satisfaction) of AutoFOCUS among autologous HCT caregivers through measurable benchmarks. We also explored changes in psychological measures, as well as engagement with the daily mindfulness strategies, meditations, and daily assessments completed through the smartphone, which were novel aspects of AutoFOCUS.

## Methods

### Recruitment and participants

Caregiver eligibility was assessed based on the following inclusion criteria: (1) ≥18 years of age; (2) being the primary caregiver for a patient scheduled to receive an autologous HCT at Moffitt Cancer Center; (3) intending to remain as the primary caregiver throughout the patient’s treatment; (4) able to speak, read, and write in English; (5) able to provide informed consent; and (6) owning a smartphone and willing to download the smartphone app. Our target sample size was determined based on recommendations for feasibility/acceptability studies (Amy et al. 2016; Faulkner 2003) and our previous work among allogeneic caregivers (Vinci et al. 2020). Benchmark criteria for recruitment, retention, and session attendance were informed by prior literature (Bowen et al. 2009; Freedland 2020; Pfledderer et al. 2024) and by outcomes observed in our previous FOCUS intervention pilot study among HCT caregivers (Vinci et al. 2020). Recruitment started in October 2022 and ended in April 2023. The study was approved by the Institutional Review Board of Advarra (IRB #00000971). This study was completed in accordance with the ethical standards as described in the 1964 Declaration of Helsinki.

## Procedures

### Screening, consent, and assessment

An initial review of scheduled autologous transplants identified potential participants. Once identified, study staff contacted potentially eligible caregivers by phone to explain the study and confirm eligibility. Eligible caregivers provided verbal informed consent and were scheduled for 6 telehealth sessions. They received an online baseline survey via email and/or text message, and a mailed package containing a treatment booklet, headphones, a box of raisins (for the first meditation session), a paper consent form, and a list of support groups/resources available at the cancer center.

After session 6, caregivers completed an end-of-treatment survey 1 week later and a follow-up survey 1 month later. Follow-up surveys were sent by text and/or email via an online link. All participants were contacted to complete a brief phone interview with a staff member. Caregivers were compensated for completing the assessment measures and received $25 for completing each survey at baseline, end of treatment, and 1-month follow-up (up to $75). For completing the phone interview at the 1-month follow-up, caregivers were compensated $25.

### Treatment sessions

Unlike our original FOCUS intervention, which included in-person and telehealth sessions, all AutoFOCUS sessions were provided via telehealth over the Zoom video conferencing platform. Sessions took place 1-on-1 with a trained facilitator over 6 consecutive weeks. Caregivers were consented about 21 days prior to transplant. Session 1 typically occurred about 2 weeks pre-transplant.

The intervention session content (see Supplemental Table 5) was systematically developed and informed by both the extant literature on mindfulness and cancer caregivers (Carmody et al. 2009; Creswell and Lindsay 2014; Grabovac et al. 2011; Hölzel et al. 2011), as well as feedback from allogeneic HCT caregivers and HCT staff (Vinci et al. 2020). Some content was updated to further tailor the intervention to autologous caregivers (e.g., removing references to graft-versus-host disease; excluding the session topic on preparing for discharge for caregivers of outpatient transplant patients). During each session, a trained facilitator guided the caregiver through about 2 formal mindfulness meditations, each lasting around 10 min. The first 3 sessions focused on training caregivers to direct their attention to their present-moment experiences, such as their breath and other physical sensations. The last three sessions encouraged caregivers to apply these skills to their thoughts and emotions. At the end of each session, the facilitator reviewed relevant mindfulness exercises for the upcoming week, and the caregiver was instructed on how to complete the home practice, which consisted of completing formal meditations, mindfulness strategies, and a daily assessment through the app (see “Daily assessment” section). All sessions were audio recorded to assist with facilitator supervision and treatment fidelity.

### Smartphone app

In addition to the telehealth sessions, participants used the LifeData RealLife EXP mobile app (lifedatacorp.com) to access meditation audio recordings, practice brief mindfulness strategies, and complete daily assessments. The app content was specifically developed to supplement the AutoFOCUS program and was informed by our prior FOCUS intervention for allogeneic HCT caregivers, relevant mindfulness literature, and feedback from HCT caregivers and clinical staff (Carmody et al. 2009; Hölzel et al. 2011; Vinci et al. 2020). At the end of Session 1, facilitators assisted caregivers in downloading and accessing the study app. A daily reminder to listen to the audio meditations on the app was sent at 9 am, which included the links to the meditations for that week. Each week, caregivers were assigned formal meditation practices that were introduced during the Zoom sessions and made available through the study app. Participants were encouraged to complete these assigned meditations independently between Zoom sessions. Daily brief mindfulness strategies were also randomly sent once per day and drawn from a pool of 96 strategies (i.e., 83 mindfulness strategies and 13 motivational messages). The mindfulness strategies fell into the following content areas: focusing on the breath, noticing thoughts, awareness of sensations, acceptance/non-judgement, gratitude, lovingkindness, and uncertainty. The intention of prompting these strategies through the app was to enhance and reinforce the use of mindfulness in daily life. Participants took between 1 and 3 min to engage in these strategies per day. Some examples include statements such as: “Emotions can change from moment to moment. Notice how you’re feeling right now.”; “Notice if you want things to be different than they are right now. Try to be present, without trying to change anything.”; “Over the next several minutes, observe your thoughts and notice if you can be kind to yourself.”

Every evening during the 6 weeks, caregivers received a daily assessment prompt via the app to answer 10 questions to assess mindfulness, affect, and self-efficacy (i.e., a total of 50 prompts were sent over 6 weeks). Caregivers selected a time between 5 and 8 pm to receive the daily assessment prompts.

## Measures

The primary outcomes of this study were feasibility and acceptability (see Table 1 for definitions and measurable benchmarks). Self-reported demographic variables (e.g., gender, age, race/ethnicity) were collected at baseline. Engagement in any support groups or other psychological treatment received by caregivers was also collected.

**Table 1.** Measurable benchmarks

	Benchmark	Expected outcome	Study result
Feasibility	Recruitment	Recruit 4 participants per month	5 participants per month were recruited
	Retention	≥80% of participants will complete the study through follow-up	84.6% of participants completed the study through follow-up
	Session Attendance	≥35% of participants will complete 100% of AutoFOCUS Sessions ≥50% will complete between 50% and 75% of AutoFOCUS Sessions	53.8% of participants completed 100% of AutoFOCUS Sessions 73.1% completed at least 67% (i.e., 4) Sessions
Acceptability	Participant Satisfaction	Client Satisfaction Questionnaire (satisfaction of ≥80%)	94.7% participants rated AutoFOCUS as ≥3 (out of 4) (M = 3.56; SD = 0.43)
	Intention to continue to use the skills learned from the program	Questionnaire developed for this study (≥8 on a 10-point Likert scale)	8.58 (SD = 1.81)

*Note*: AutoFOCUS = Focusing On mindfulness for Caregivers Under Stress, (M = mean, SD = standard deviation).

### Questionnaires

Caregiver burden was assessed by the 12-item Zarit Burden Interview Short Form, which uses a 5-point Likert scale (Bédard et al. 2001). Depression was assessed by the 20-item Center for Epidemiological Studies Depression Scale, which uses a 4-point Likert scale (Lenore 1977). Anxiety was assessed by the 7-item Generalized Anxiety Disorder-7, which uses a 4-point Likert scale (Spitzer et al. 2006). Overall distress was assessed by the 1-item National Comprehensive Cancer Network Distress Thermometer (Panel 1999). Stress was assessed by the 14-item Perceived Stress Scale, which uses a 5-point Likert scale (Cohen et al. 1983). Affect was assessed by the 20-item Positive and Negative Affect Schedule, which uses a 5-point Likert scale (Watson et al. 1988). Higher scores on all the measures above indicate greater levels of each construct.

Post-traumatic growth was assessed by the 21-item Post Traumatic Growth Inventory (PTGI), which uses a 6-point Likert scale (Tedeschi and Calhoun 1996). The PTGI assessed 5 factors: Relating to Others, New Possibilities, Personal Strength, Spiritual Change, and Appreciation of Life. Mindfulness was assessed by the 15-item Mindful Attention Awareness Scale, which uses a 6-point Likert scale, and the 15-item Five Facet Mindfulness Questionnaire- Short Form, which uses a 5-point Likert scale (Brown and Ryan 2003). Self-efficacy was assessed by the 21-item Caregiver Self-efficacy Scale (CaSES) which uses a 4-point Likert scale (Ugalde et al. 2013).

### Caregiver engagement with home practice

To assess caregiver engagement with the home practice, completion of the meditations and mindfulness strategies was monitored via the app. We also asked participants to report on the amount of time they spent each day meditating at the end-of-treatment assessment.

### Treatment feedback

The end-of-treatment assessment included questions to obtain caregiver feedback on their experience with AutoFOCUS. The 8-item Client Satisfaction Questionnaire (Larsen et al. 1979) was used to measure participant satisfaction. Several other treatment-specific questions were developed by the study team, including a 4-item multiple-choice self-report measure to assess participant’s feedback on the number, timing, and content of strategies sent by the app. A 3-item, 5-point Likert scale measure was also developed to obtain feedback on the ease/difficulty and convenience of using the app.

Participants also completed an in-depth phone interview where they were asked about their perceptions of the treatment content; any logistical issues they encountered as part of the treatment; barriers to engaging in treatment; as well as their feedback on participating in the sessions over Zoom.

### Daily assessment

A 10-item daily assessment measured state mindfulness (i.e., attention, nonjudgment/acceptance, decentering) via 3 items selected from existing self-report measures of mindfulness (Baer et al. 2006; Brown and Ryan 2003; Lau et al. 2006), state affect (i.e., stressed, overwhelmed, frustrated, drained, guilty, hopeful),(Watson and Clark 1999) and self-efficacy (i.e., “I have confidence in my ability to take care of my patient”) (Ugalde et al. 2013). Daily assessments were included to evaluate the feasibility participants completing frequent, repeated measurements of mindfulness, affect, and self-efficacy during the intervention.

## Data analysis

Although the sample was not powered to conduct inferential statistics, descriptive analyses and changes from pre- to post-intervention via paired sample *t*-tests on all questionnaires were examined. Cohen’s *d* was used to report effect sizes [ES] (*d* = 0.2 small, *d* = 0.5 medium, *d* = 0.8 large). Caregiver interviews at the 1-month follow-up were audio-recorded and transcribed by study staff prior to thematic analysis using NVivo in which key themes were derived.

## Results

**Figure 1.**
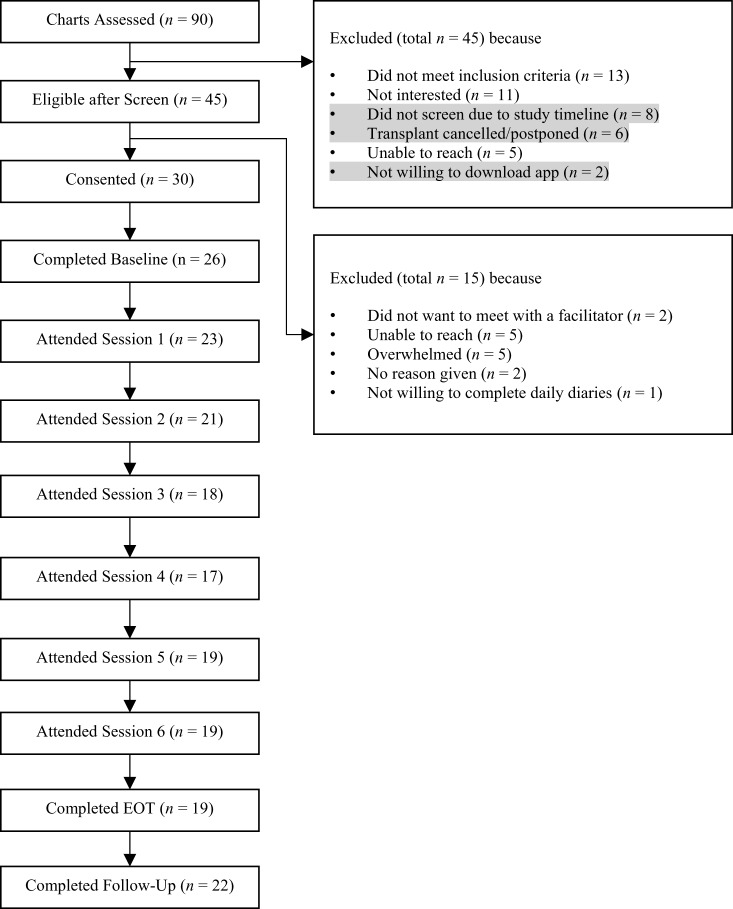
CONSORT diagram.

Figure 1 presents the CONSORT flow diagram of the study. An average of 5 participants consented to participate each month. Forty-five caregivers were deemed eligible per phone screening, and 30 consented to participate. The number of inpatient vs. outpatient caregivers that consented was monitored for equal representation in the study (i.e., enrollment goal: 15 inpatient and 15 outpatient caregivers). Due to this, 8 outpatient caregivers were deemed ineligible after the maximum number of outpatient caregivers were met. Of the 30 who consented, 26 completed the baseline. Among these 26, 19 (73.1%) completed at least 4 sessions, and 14 (53.8%) completed all 6 sessions. Twenty-two participants (84.6%) completed the study through follow-up. Together, our benchmarks for recruitment, retention, and session attendance, as presented in Table 1, were met. Table 2 presents the demographics of the sample (e.g., 88.5% female; 7.7% Hispanic/Latinx; 11.5% Black/African American).

**Table 2.** Sample characteristics at baseline

Variable (*N* = 26)	Mean (SD) or *N* (%)
Caregiver Type	
Inpatient transplant	*N* = 9
Outpatient transplant	*N* = 17
Age	57.7 (9.6)
Gender: Female	23 (88.5%)
Sexual Orientation	
Straight/Heterosexual	25 (96.2%)
Bisexual	1 (3.8%)
Ethnicity: Hispanic/Latinx	2 (7.7%)
Race (check all that apply)	
White	18 (69.2%)
Black/African American	3 (11.5%)
Asian	2 (7.7%)
Other	3 (11.5%)
Marital Status	
Married/Partnered/Living with significant other	25 (96.1%)
Divorced	1 (3.8%)
Relation with the patient	
Spouse/Partner	19 (73.1%)
Parent	5 (19.2%)
Sibling	1 (3.8%)
Child	1 (3.8%)
Education	
High school diploma and/or GED	5 (19.2%)
Some college, no diploma	5 (19.2%)
Associate degree	4 (15.4%)
Undergraduate degree	4 (15.4%)
Graduate degree	8 (30.8%)
Employment status (currently as a caregiver)	
Full-time work (≥40 h per week)	8 (30.8%)
Part-time work (<40 h per week)	3 (11.5%)
Homemaker	2 (7.7%)
Retired	11 (42.3%)
Unemployed, unable to work, or disabled	2 (7.6%)
Annual Household Income	
<$40,000	7 (26.9%)
$40,000–$59,999	6 (23.1%)
$60,000–$79,999	4 (15.4%)
$100,000–$159,999	4 (15.3%)
≥$160,000	2 (7.7%)
I choose not to answer	3 (11.5%)
Current Use of Mental Health Services (could select more than 1)	
Support group	1 (3.8%)
Individual counseling	5 (19.2%)
Prescribed medication	6 (23.1%)
Smartphone app	1 (3.8%)
None	18 (69.2%)

Regarding acceptability (see Table 3), participants reported high satisfaction and intent to continue using the skills learned. Overall, the perceived ease of use of the app was high. Over one-third of participants reported that the number of mindfulness strategies sent each day was “just right,” and they also found the content variety to be appropriate. Over 50% liked the amount and random delivery of the mindfulness strategies (although about 42% reported the strategies sometimes arrived at inconvenient times). The overall perceived usefulness of mindfulness strategies was also high.

**Table 3.** Acceptability outcomes

	Mean (SD) or *N* (%)	
Variable	EOT (*N* = 19)	Follow-up (*N* = 22)
Perceived Easiness of App Use		
Perceived Difficulty of Using the App (1 = Very difficult, 6 = Very easy)		
At the beginning of the study	5.11 (1.20)	-
By the end of the study	5.68 (0.67)	-
Perceived Convenience of Accessing Meditations Through the App (1 = Very convenient, 6 = Not at all convenient)	1.58 (1.07)	-
Perceived Appropriateness of the Intervention Content through App		
Number of Mindfulness Strategies Sent Each Day		
Would have liked more strategies	2 (10.5%)	-
The number of strategies was just right	14 (73.7%)	-
Would have liked fewer strategies	3 (15.8%)	-
Number of Mindfulness Strategies Sent Over Time^a^		
Liked the consistent amount	11 (57.9%)	-
Would have like more strategies over time	2 (10.5%)	-
Would have liked fewer strategies over time	5 (26.3%)	-
Timing of the Mindfulness Strategies Received		
Liked the random strategies	10 (52.6%)	-
Sometimes felt the strategies arrived at inconvenient times	8 (42.1%)	-
Often felt the strategies arrived at inconvenient times	1 (5.3%)	-
Perceived Variety in Content of the Mindfulness Strategies		
Too much variety	1 (5.3%)	-
The variety was just right	16 (84.2%)	-
Would have liked more variety	2 (10.5%)	-
Perceived Usefulness of Mindfulness Strategies (1 = Not at all useful, 10 = Very useful)	8.00 (2.13)	-
Self-Reported App Use to Practice Mindfulness and Continued Practice		
Average Number of Days/Week Mindfulness Strategies Completed		
1−2 days	8 (42.1%)	7 (31.8%)
3−4 days	2 (10.5%)	9 (40.9%)
5−6 days	4 (21.1%)	3 (13.6%)
Everyday	5 (26.3%)	1 (4.5%)
Never	-	2 (9.1%)
Average Number of Days/Week Practicing Meditations^a^		
1−2 days	7 (36.8%)	13 (59.1%)
3−4 days	4 (21.1%)	4 (18.2%)
5−6 days	4 (21.1%)	2 (9.1%)
Everyday	3 (15.8%)	1 (4.5%)
Never	-	2 (9.1%)
Time Spent Practicing Meditation Each Time^a^		
<5 min	1 (5.3%)	-
10 min	12 (63.2%)	-
15 min	4 (21.1%)	-
>15 min	1 (5.3%)	-

*Note*: EOT = end of treatment.

a *n* = 1 missing due to incomplete responses.

Among the 26 participants who completed the baseline, 23 downloaded the app after completing Session 1 and were included in our subsequent analyses of app use data. All data reported in this paragraph were derived from app metadata (i.e., not self-report). On average, participants completed 20.13 (SD = 16.06) daily assessments over the 6 weeks. Eleven individuals (47.8%) completed at least half of the daily assessments that they received. Participants completed on average 15.61 (SD = 14.65) brief mindfulness strategies. Seven participants (30.4%) completed at least half of the strategies that they received. Regarding the reminders to complete meditations sent over the 6 weeks, 16 participants (69.6%) completed at least 1 meditation in immediate response to notifications. Of these 16 participants, 9 completed at least 3 meditations in response to notifications over the 6 weeks. Some participants (*n* = 10; 43.5%) also completed at least 1 meditation using the on-demand feature. Altogether, 6 participants (26.1%) did not complete any meditations via the app, 2 participants (8.7%) completed 1 meditation, 7 participants (30.4%) completed 2–5 meditations, 3 participants (13.0%) completed 6–10 meditations, and the remaining 5 participants (21.7%) completed more than 20 meditations over the 6-week period.

Regarding self-reported frequency of mindfulness practice on the end-of-treatment assessment, over one-third of participants self-reported practicing mindfulness strategies and meditations at least 5 days per week, whereas only a few continued to practice on most days per week at follow-up (see Table 3). Many participants reported spending at least 10 min when practicing the meditations. Common barriers included (1) being unable to find time, (2) forgetting about it, and (3) needing to attend to their patients. Six participants (23.1%) reported downloading the meditations to their personal devices, indicating that they intended to use them outside of the app in the study context. Participants reported the following meditations as the most helpful and enjoyable: (1) Sitting Meditation on the Breath; (2) Meditation on Thoughts; and (3) Mindful Stretching. Participants found that the Walking Meditation was the least helpful and enjoyable.

## Questionnaires

As shown in Table 4, from baseline to end of treatment, there were significant decreases in overall distress (*p* < .001, ES = 0.99), anxiety (*p* = .032, ES = 0.53), perceived stress (*p* = .035, ES = 0.52), and negative affect (*p* = .008, ES = 0.69), and a significant increase in overall post-traumatic growth (*p* = .009, ES = 0.67) with medium to large ES observed across these outcomes. These results were maintained through the 1-month follow-up.

**Table 4.** Descriptive statistics of self-report measures and *t*-test results

	M (SD)			Effect size		
Variable	Baseline (*N* = 26)	EOT (*N* = 19)	Follow-up (*N* = 22)	Baseline – EOT	Baseline – Follow-Up	EOT – Follow-up
AFFECT/STRESS						
Distress Thermometer	5.38 (2.94)	3.11 (2.54)	3.27 (2.41)	0.99*	0.67**	0.07
CESD Total Score	16.71 (16.54)^a^	12.58 (10.65)	12.80 (12.29)^c^	0.28	0.21	0.04
GAD-7 Total Score	7.69 (6.24)	5.11 (4.43)	4.18 (4.38)	0.53***	0.74**	0.34
PSS Total Score	22.92 (9.40)	19.37 (7.33)	18.86 (7.85)^b^	0.52***	0.64**	0.18
PANAS						
PANAS Positive Affect	33.62 (9.03)	35.84 (7.40)	35.41 (8.56)	0.24	0.18	0.09
PANAS Negative Affect	21.73 (7.90)	16.68 (6.36)	16.10 (5.65)^c^	0.69**	0.88**	0.38
MINDFULNESS						
MAAS Total Score	4.53 (1.05)	4.61 (0.83)	4.56 (0.92)^b^	0.03	0.04	0.13
FFMQ-15 Nonjudging	12.31 (2.24)	12.26 (2.40)	12.57 (2.11)^b^	0.05	0.16	0.29
COPING						
ZBI Total Score	11.65 (8.96)	11.11 (7.59)	10.86 (8.09)	0.32	0.29	0.01
PTGI Total Score	45.46 (24.52)	68.84 (24.39)	65.09 (25.16)	0.67**	0.64**	0.06
PTGI Relating to Others	16.65 (8.80)	24.00 (9.27)	22.68 (8.87)	0.49***	0.48***	0.03
PTGI New Possibilities	7.85 (5.91)	12.95 (6.65)	12.05 (6.46)	0.60***	0.59***	0.00
PTGI Personal Strength	8.35 (5.64)	14.11 (4.81)	13.18 (5.22)	0.77**	0.72**	0.10
PTGI Spiritual Change	4.31 (3.66)	6.42 (3.66)	6.64 (3.46)	0.52***	0.54***	0.02
PTGI Appreciation of Life	8.31 (4.23)	11.37 (2.97)	10.55 (4.06)	0.75**	0.51***	0.14
CASES						
CASES Resilience	3.71 (0.37)	3.48 (0.59)	3.41 (0.74)^c^	0.43	0.43	0.03
CASES Self-Maintenance	3.12 (0.79)	3.24 (0.73)	3.13 (0.66)^c^	0.21	0.14	0.06
CASES Emotional Connectivity	3.75 (0.41)	3.77 (0.26)	3.58 (0.43)^c^	0.02	0.38	0.28
CASES Instrumental Caregiving	3.53 (0.45)	3.58 (0.39)	3.64 (0.36)^c^	0.03	0.32	0.39

*Note*: EOT = end of treatment; CESD = Center for Epidemiological Studies Depression; GAD-7 = General Anxiety Disorder-7; PSS = Perceived Stress Scale; PANAS = Positive and Negative Affect Schedule; MAAS = Mindful Attention Awareness Scale; FFMQ = Five Facet Mindfulness Questionnaire; ZBI = Zarit Burden Interview; PTGI = Post Traumatic Growth Inventory; CASES = Caregiver Self-Efficacy Scale. Effect sizes were categorized as small (*d* = 0.2), medium (*d* = 0.5), and large (*d* = 0.8) according to Cohen (1988).

a *n* = 5 missing due to incomplete responses.

b *n* = 1 missing due to incomplete responses.

c *n* = 2 missing due to incomplete responses.

* *p* < .001, **p < .01, ***p < .05.

## Intervention feedback

Participant feedback from follow-up interviews generally reflected a positive perception of the intervention (see Supplemental Table 6). Participants preferred starting the intervention before their patient’s transplant.

Most participants found the meditations easily accessible through the study app and appreciated reminders, although some wanted more flexibility in choosing the time for the meditation reminders to better align with their schedules. Barriers to listening to the meditations included patient care responsibilities and scheduling constraints. Overall, participants found the app easy to navigate. Participants had a favorable view of the daily mindfulness strategies, expressing no desire to alter their frequency. The participants reported positive experiences with Zoom sessions, noting that it was convenient and facilitated connections with facilitators. Feedback between inpatient and outpatient caregivers were consistent with no notable differences.

## Discussion

The current study demonstrated high feasibility and acceptability of an MBI among autologous caregivers, called AutoFOCUS. This study utilized a novel approach by supplementing the face-to-face telehealth intervention with a study-specific app. Overall, results from this study provide insight into the capabilities of caregivers of autologous HCT patients to complete an MBI amidst the challenges of caring for patients undergoing HCT transplants.

### Implications

This study established high feasibility and acceptability of delivering an innovative MBI intervention to caregivers of autologous HCT patients via telehealth. AutoFOCUS may be an appropriate intervention to address the unique needs of autologous caregivers to reduce stress to aid in patient health outcomes. Through the use of technology, AutoFOCUS can address the limitations of accessibility to interventions for caregivers by combining live telehealth mindfulness sessions with a smartphone app, providing caregivers access to daily mindfulness strategies and content.

Observed decreases in distress, anxiety, perceived stress, and negative affect from baseline to end of treatment align with previous findings showing that behavioral interventions reduce psychological distress in caregivers (Vinci et al. 2020). Despite caregiving challenges, participants reported a significant increase in overall post-traumatic growth from baseline to end of treatment, maintained at 1-month follow-up, indicating sustained benefits of AutoFOCUS. These findings are consistent with previous findings on post-traumatic growth, showing that coping programs can have lasting effects on psychological well-being (Tedeschi and Calhoun 1996). Our population of caregivers reflects existing literature, which shows that caregiving roles are predominantly undertaken by women and that being female is a risk factor for increased caregiver distress (Beattie and Lebel 2011). Despite consistency with our findings and the broader literature, the absence of a nontreatment comparison limits conclusions about the MBI-exclusive effects.

No significant changes emerged over time in depression, mindfulness, burden, and self-efficacy, although means trended as expected, with decreases in depression and burden and increases in mindfulness and self-efficacy. Although the intervention focused on cultivating mindfulness skills, no significant changes were observed in mindfulness measures. This may reflect limited statistical power, a need for longer practice periods, or issues considered to be inherent in the measurement of self-reported mindfulness (e.g., one’s ability to accurately rate oneself prior to fully understanding the meaning of the items/completing a mindfulness training or program) (Bergomi et al. 2013; Grossman 2011; ). Future interventions may consider extended follow-up periods, and a larger sample size to help clarify the impact of AutoFOCUS on these factors.

Feasibility of AutoFOCUS was high, surpassing our benchmark criteria of recruiting 5 participants per month, 85% retention, and 54% of participants completing all sessions (73% completing most sessions). At the end of the study, participants indicated high intention to continue to use the skills learned from the program. Participant satisfaction with the intervention was high, and feedback on the intervention aligned with app metadata, emphasizing push notifications’ role in facilitating participant engagement.

Compared to our previous pilot study with allogeneic HCT caregivers, this study showed similar outcomes, demonstrating the feasibility and acceptability of an MBI for both autologous and allogeneic HCT caregivers (Vinci et al. 2020). Both studies observed significant decreases in negative affect and significant increases in post-traumatic growth from baseline to end of treatment. Intervention feedback demonstrated similar levels of participant satisfaction, particularly with the frequency of home practice meditations, the perceived usefulness of the treatment, and engagement with the mindfulness skills following the treatment. The consistency in results across both studies underscores the potential benefit of FOCUS as an intervention for addressing the needs of HCT caregivers across different patient care contexts.

Findings on app engagement highlight the potential benefit of integrating a smartphone app with face-to-face intervention content, to encourage daily mindfulness practice. Higher engagement in meditations after push notifications suggests this feature likely facilitated practice. Encouraging daily mindfulness practice is important, as consistent engagement is associated with strengthening key mechanisms of mindfulness interventions, including enhanced attentional control, improved emotion regulation, and greater acceptance of present-moment experiences (Creswell and Lindsay 2014; Hölzel et al. 2011). Supporting regular practice, by providing easy access to audio recorded meditations, may therefore be an important factor in achieving and sustaining intervention-related benefits. Participants reported liking the daily mindfulness strategies sent via the app, indicating the potential of real-time intervention in this population. Participant feedback on the number and frequency of notifications also highlights the acceptability of this approach amongst this population.

Daily assessment data collected over 6 weeks provides insight into its feasibility among this caregiving population. The inclusion of daily assessments over 50 days represents a novel feasibility finding, demonstrating that caregivers were willing and able to complete repeated self-reports over an extended timeframe during a high-burden caregiving experience. This supports the potential for future studies to capture dynamic, real-time changes in psychological constructs such as affect, stress, and mindfulness across the transplant trajectory. This type of data can inform the development of momentary interventions, including just-in-time adaptive strategies, which are particularly relevant for populations facing rapidly shifting emotional and practical demands. Until recently, only 1 study used daily assessments for caregivers of HCT patients, indicating initial feasibility and acceptability (Kroemeke et al. 2019). Our study extends work in this area by demonstrating the feasibility of collecting daily assessments from HCT caregivers over a longer period (50 vs. 28 days) spanning pre-transplant through post-discharge.

## Study limitations

Future research is needed to assess the efficacy of this intervention on a larger scale through a randomized clinical trial with a comparison condition, examining both caregiver and patient outcomes. Consistent with the recommended sample size for pilot studies (Amy et al. 2016; Faulkner 2003; Vinci et al. 2020), our sample size was chosen for assessing the feasibility and acceptability of this MBI and the design was a single arm. The sample primarily consisted of non-Hispanic White female individuals, highlighting the need for testing this intervention among a more racially and ethnically diverse sample along with a greater representation of male caregivers.

## Significance of results

AutoFOCUS is a novel MBI designed to reduce autologous HCT caregiver distress and potentially improve patient outcomes. This study highlights the feasibility and acceptability of this intervention, and qualitative feedback underscoring the overall positive perception of AutoFOCUS by HCT caregivers.

## Supporting information

10.1017/S1478951525100485.sm001 Yepez et al. supplementary material Yepez et al. supplementary material

## Supplementary material

The supplementary material for this article can be found at https://doi.org/.10.1017/S1478951525100485.
