# Supplementary material for: AutoFOCUS: A mindfulness-based intervention for caregivers of autologous hematopoietic stem cell transplant recipients
Source: Palliat Support Care. 2025 Aug 22;23:e147. doi: 10.1017/S1478951525100485 (PMC12491394; doi:10.1017/S1478951525100485)
Supplement: Yepez et al. supplementary material [file S1478951525100485sup001.docx]

**AutoFOCUS: A mindfulness-based intervention for caregivers of autologous hematopoietic stem cell transplant recipients**

Valerie Yepez^1^, Min-Jeong Yang^2^, Sierra Washington^2^, Sarah Jones^2^, Ranjita Poudel^2^, Joseph Pidala^3^, Marilyn Horta^2^, & Christine Vinci*^2,4^

^1^Philadelphia College of Osteopathic Medicine South Georgia, Moultrie, GA, United States of America

^2^Department of Health Outcomes and Behavior, H. Lee Moffitt Cancer Center, Tampa, FL, United States of America

^3^Department of Blood and Marrow Transplantation and Cellular Immunotherapy, H. Lee Moffitt Cancer Center, Tampa, FL, United States of America

^4^Department of Oncological Science, College of Medicine, University of South Florida, Tampa, FL, United States of America

Corresponding Author: Christine Vinci, [christine.vinci@moffitt.org](mailto:christine.vinci@moffitt.org)

**Table 5**

***Session Content***

| **Session** | **Topic** | **Discussion Topics/Activities** | **In-Session Formal Meditation** | **Weekly Practice** |
| --- | --- | --- | --- | --- |
| **1** | Mindfulness for Caregivers | - Existing coping strategies - Introduction to mindfulness - Doing mode vs Being mode | - Raisin exercise (5 min) - Mindful stretching (10 min) - Sitting meditation: breath (10 min) | - Sitting meditation: breath (daily, 10 min) - Mindful stretching (daily, 10 min) - Engage in one coping strategy over the next week |
| **2** | Awareness of Stressors and the Experience of Stress | - Identification of current and anticipated stressors - How does stress show up in the body? - Surfing the Stress | - Sitting meditation: breath (10 min) - Body scan (20 min) | - Body scan (daily, 15 min) - Either sitting meditation: breath or mindful stretching (daily, 10 min) - Awareness of how stress impacts the body |
| **3** | Skillful Action | - Uncontrollability vs skillful action - Taking time to care for yourself - STOP (Stop, Take a Breath, Observe, Proceed) - Preparing for discharge | - Sitting meditation: senses (10 min) - Mountain meditation (7 min) | - Sitting meditation: senses (daily, 10 min) - Body scan or mindful stretching (daily, 10-15 min) - STOP - Preparing for discharge^a^ – read through caregiving class notes and list any questions for nursing staff |
| **4** | Thoughts are Thoughts | - Discussion of thoughts as related to emotions and physical pain - Evaluation of self as caregiver | - Present moment awareness (5 min) - Sitting meditation: thoughts (10 min) - Walking meditation (10 min) | - Walking meditation (daily, 10 min) - Sitting meditation: thoughts (daily, 10 min) |
| **5** | Self-care and Balance | - Pleasant activities - Taking care of the self | - Gratitude practice (10 min) - Loving-kindness meditation (20 min) | - Engage in pleasant activities daily - Loving-kindness meditation (daily, 20 min) |
| **6** | Planning for the Future | - What to do if things change (for better or worse) - Continuing to integrate mindfulness into daily life | - Caregiver’s choice (10 min) - Sitting meditation: breath (10 min) |  |

Note. ^a^Applies only to caregivers of inpatient autologous transplant patients.

**Table 6**

***Intervention Feedback***

|  | **Question** | **Derived Category/Theme** | | **Quotes** |  |
| --- | --- | --- | --- | --- | --- |
| **General** | Reason to participate in study | - Anticipated stress - Curiosity - Patient care | | - "I knew this time around it was going to be a little bit harder and so I reached out to Moffitt. I don’t know how I got involved in this but I’m so thankful that someone picked my name or gave it to me that, I- I think everyone should have this service " 28, AGE 58, F, Inpatient - "I wanted to see what the, what the study was about." 20, AGE 48, F, Inpatient - "I know these studies and focus groups and everything else can really help improve care that we give.” 59, AGE 67, F, Outpatient |  |
| **FOCUS Intervention** | Thoughts on timeline of sessions | | - Having sessions before the transplant | - "I think I will probably say earlier than it started. That’s my perspective. I had more difficulty before we even got to Moffitt dealing with his diagnosis than when I got to Moffitt and I was with him." 59, AGE 67, F, Outpatient | |
|  | Treatment Session schedule and length of time | - Intervention schedule satisfaction - Shorter amount of sessions or length of time | | - “Having it [sessions] the same time every week was perfect.” 28, AGE 58, F, Inpatient - "I think that 45 minutes to an hour was too long. Maybe around 30 minutes." 54, AGE 74, M, Inpatient |  |
|  | Able to meditate as often as you want | - Barriers - Facilitators | | - **Patient Care and Scheduling** “I couldn't really get out per say because I couldn't leave him too long, so when we were there, it was really tough.” 73, AGE 58, F, Inpatient |  |
|  |  |  |  | - **Helpful Reminders** "[Reminders] I wouldn’t want to say forced me to do it, but it encouraged me to do it so that I would see if it helped me or not." 28, AGE 58, F, Inpatient - **Enjoyment** "I actually miss having the app. Plus having the app, you know that you can go online and find different meditations." 51, AGE 56, F, Outpatient |  |
| **Smartphone App** | Thoughts on accessing meditations | - Easy to access | | - "Everything was very convenient, it was a simple app nothing to complicate it." 69, age 60, F, Inpatient |  |
|  | 9 am meditation reminder | - Flexibility in choosing the time | | - If I could have more ownership of that it probably would have been more helpful for me.” 32, AGE 49, F, Inpatient - "The reminders were very necessary so that, that was also what kept me continuing to do the mindful meditations." 20, AGE 48, F, Inpatient |  |
|  | Usability of app | - Easy to use | | - " Your app is very easy then you can click wherever you want to go.” 14 F, AGE 72, Outpatient |  |
|  | Mindful strategies | - Helpful and positive feedback regarding timing and number sent | | - "They were helpful." 54, AGE 74, M, Inpatient - "I don't think we got too many throughout the day to where it was annoying at all." 20, AGE 48, F, Inpatient - "They were short, they were helpful." 54, AGE 74, M, Inpatient |  |
| **Zoom** | Experience meeting over Zoom | - General Satisfaction - Convenience - Interpersonal connection | | - "It was very convenient, you know, it would've been really hard to try to do that in person." 59, AGE 67, F, Outpatient - "It's comfortable, and I didn't have any issues meeting through zoom at all." 32, AGE 49, F, Inpatient - "It's like being right there in the room with her." 81, AGE 67, F, Outpatient |  |
|  | Difficulties meeting over Zoom | - Very few challenges noted | | - "It actually made it easier for me." 39, AGE 58, M, Inpatient - "I remember thinking to myself ‘this works good when he's not here’ because I have privacy but there was one time I think that didn't work out so well." 59, AGE 67, F, Outpatient - "There was like maybe once, you know how like you can't get your volume turned up." 73, AGE 58, F, Outpatient |  |
|  | Feedback on meeting in-person vs remote |  | | - **Time** “I know everybody's schedule is very busy, I really didn't have the need to physically meet in person, the Zoom calls would suffice." 69, AGE 60, F, Inpatient - **Isolation Precautions** "We had to isolate, so that worked out with this situation for sure." 8, AGE 53, F, Inpatient - **Patient Care** "I probably would've been more distracted if I was there actually, cause with Zoom I was in a room where I was, it was quiet, and I didn't have to worry about him." 81, AGE 67, F, Outpatient - **No change** "No, I think it was fine the way it was." 55, AGE 48, F, Outpatient - **Personal Preference** "I wouldn't have minded that. I'm a people person though so." 73, AGE 58, F, Outpatient |  |
